# Appearance and Prevalence of JN.1 SARS-CoV-2 Variant in India and Its Clinical Profile in the State of Maharashtra

**DOI:** 10.7759/cureus.56718

**Published:** 2024-03-22

**Authors:** Rajesh P Karyakarte, Rashmita Das, Mansi V Rajmane, Sonali Dudhate, Jeanne Agarasen, Praveena Pillai, Priyanka M Chandankhede, Rutika S Labhshetwar, Yogita Gadiyal, Preeti P Kulkarni, Safanah Nizarudeen, Sushma Yanamandra, Nyabom Taji, Suvarna Joshi, Varsha Potdar

**Affiliations:** 1 Microbiology, Byramjee Jeejeebhoy Government Medical College and Sassoon General Hospitals, Pune, IND; 2 Microbiology, Byramjee Jeejeebhoy Government Medical College, Pune, IND; 3 Infectious Diseases, Indian Council of Medical Research, National Institute of Virology, Pune, IND; 4 Pediatrics, Byramjee Jeejeebhoy Government Medical College, Pune, IND

**Keywords:** covid-19, severe acute respiratory syndrome coronavirus 2, sars-cov-2, clinical characteristics, covid-19 disease, pirola, ba.2.86, ba.2.86.1.1, jn.1

## Abstract

Background: In August 2023, the BA.2.86 SARS-CoV-2 variant, with over 30 spike protein mutations, emerged amidst the global dominance of XBB sub-lineages. It evolved into JN.1 by late 2023, spreading across 71 countries. JN.1, distinct for its L455S mutation, significantly dominated global sequences, raising concerns over its transmission and clinical impact. The study investigates JN.1's clinical severity and its effect on hospital admissions in Maharashtra, India.

Methodology: The present study involved 3,150 curated Indian SARS-CoV-2 whole genome sequences with collection dates between 1st August 2023 and 15th January 2024. Lineage and phylogenetic analysis of sequences was performed using Nextclade. Telephonic interviews were conducted to confirm the demographic details and obtain clinical information on the JN.1* (* indicates JN.1 and all its sub-lineages) cases. The obtained data were recorded and analyzed using Microsoft® Excel (Microsoft Corporation, Redmond, WA).

Results: Out of 3,150 sequences analyzed, JN.1* was the most common lineage (2377/3150, 75.46%), followed by XBB.2.3* (281/3150, 8.92%) and XBB.1.16* (187/3150, 5.94%). In India, it was first identified on 6th October 2023, in Kerala. The highest proportion of JN.1* sequences originated from Maharashtra (628/2377, 26.42%), followed by West Bengal (320/2377, 13.46%), Andhra Pradesh (293/2377, 12.33%), Kerala (288/2377, 12.12%), and Karnataka (285/2377, 11.99%). In Maharashtra, the JN.1* variant was first identified on 23rd November 2023. A total of 279 JN.1* cases were included in the clinical study. Of these, 95.34% (266/279) had symptomatic disease with mild symptoms; cold (187/279, 67.03%) being the most common symptom, followed by fever (156/279, 55.91%), cough (114/279, 40.86%), and headache (28/279, 15.64%). Of all the cases, 13.26% (37/279) required institutional quarantine or hospitalization, and the rest were isolated at home. Among the hospitalized patients, 54.05% (20/37) cases were given conservative treatment while 45.95% (17/37) cases required supplemental oxygen therapy. Regarding the vaccination status, 94.26% (263/279) of cases received at least one dose of the COVID-19 vaccine, while 5.02% (14/279) were not vaccinated, of which most were children aged zero to nine years (5/14, 35.71%). The overall recovery rate among JN.1* cases was 98.57% (275/279), with 1.43% (4/279) cases succumbing to the disease.

Conclusion: The JN.1* variant, the dominant variant in India, exhibits clinical features similar to previous circulating variants in Maharashtra without increased severity. Its notable transmissibility underscores the importance of studying the ongoing viral evolution. The pressing necessity for swift identification and the clinical features of new variants is essential for effective public health response.

## Introduction

At the time when the sub-lineages of the recombinant SARS-CoV-2 variant, XBB, were predominant globally, a new sub-lineage of BA.2 appeared around August 2023 [1]. This new lineage of SARS-CoV-2 was labeled as BA.2.86 on 17th August 2023 [2]. BA.2.86 had more than 30 spike (S) protein mutations over and above its parent lineage, BA.2 [1]. It was first sequenced in Israel and reported on 13th August 2023 [3]. Alarmed by its potential to evade pre-existing immunity against SARS-CoV-2 developed by the administration of vaccines and from previous infections, the World Health Organization (WHO), within four days, promptly designated it as a variant under monitoring (VUM) on 17th August 2023, the day it was labeled as BA.2.86. Later, due to its growing prevalence, as evidenced by genomic surveillance, the WHO escalated its status to the variant of interest (VoI) on 21st November 2023 [4].

In late 2023, the BA.2.86 SARS-CoV-2 variant evolved, leading to the emergence of the JN.1 (BA.2.86.1.1) variant. It was first identified in the United States in September 2023 [5]. At the same time, it was identified in 12 more countries, with the highest proportions in Canada, France, Singapore, Sweden, the United Kingdom, and the United States [6]. Initially monitored within the BA.2.86 variant, the rapid and extensive spread of JN.1 across various nations prompted WHO to designate it as a VOI on 20th December 2023, thereby acknowledging its distinction from its parent lineage, BA.2.86. According to the WHO reports as of 19th January 2024, JN.1 has emerged as the most reported VOI globally, with detection in 71 countries and accounting for 65.5% of all sequences in the final week of 2023 [7]. By January 2024, the JN.1* variant dominated India, representing 97.25% of Indian sequences on the Global Initiative on Sharing All Influenza Data (GISAID), thus highlighting the significant impact and global concern over its transmission dynamics [8].

While JN.1 is closely related to its precursor, BA.2.86, it features a unique mutation, L455S, in the receptor-binding domain (RBD) of its spike protein, alongside three additional mutations in non-spike proteins. A similar L455F mutation in variants HK.3 and other FLip variants has been shown to enhance transmissibility and immune evasion compared to the original EG.5.1 variant [9]. However, information is scarce regarding the potential impact of JN.1 on clinical outcomes. Therefore, the current study aims to evaluate the clinical severity associated with JN.1 infections and its implications for hospital admissions in Maharashtra.

## Materials and methods

The present study is a part of the sequencing activity in Maharashtra under the Indian SARS-CoV-2 Genomics Consortium (INSACOG) to study the evolutionary patterns and epidemiological characteristics of SARS-CoV-2.

SARS-CoV-2 whole genome sequences in India: lineage and phylogenetic analysis

To trace the first appearance and the spread of the JN.1 SARS-CoV-2 variant in India, whole genome sequences of the virus, with sample collection dates from 1st August 2023 to 15th January 2024, submitted by various sequencing laboratories in different states and Union Territories of the country, were downloaded from the Indian Biological Data Centre (IBDC) database [10] with their kind permission. Entries with complete metadata, including geographic locations and sample collection dates, were included in the study and the sequences used are accessible in the supplemental table in the Appendices. The lineage and clade analysis were performed using Nextclade software (version 3.0.1). The phylogenetic tree was constructed using Nextclade Augur and visualized using Auspice (version 2.52.1).

Demographic and clinical data collection of SARS-CoV-2-positive cases in Maharashtra

Demographic details, including age, sex, area of residence, contact number, and date of sample collection and testing, were collected from the metadata provided to the sequencing laboratories by the reverse transcription-polymerase chain reaction (RT-PCR) testing centers. Telephonic interviews were conducted with each patient to confirm their demographic details and obtain clinical information such as symptoms, isolation type, hospitalization, oxygen requirements, treatments, and vaccination status. Those patients who chose not to disclose their clinical history during the interview were documented and excluded from the study.

Also, daily records from 1st August 2023 to 15th January 2024 were collected from the State's District Health Services Department to assess the severity of illness linked to the JN.1 SARS-CoV-2 variant during its surge in Maharashtra and its impact on public health. The data collected included the daily total number of samples tested for SARS-CoV-2, test positivity, hospital admissions, and the demand for oxygen throughout the state.

Statistical analysis

All demographic and clinical data were recorded and analyzed using Microsoft® Excel (Microsoft Corporation, Redmond, WA), and analysis was performed using Microsoft® Excel. Continuous variables were presented as median and interquartile range (IQR). Categorical variables were presented as numbers and percentages.

## Results

Distribution of SARS-CoV-2 lineages in India

A total of 3,150 downloaded sequences were included in the study following data curation. A total of 144 different lineages were identified following Nextclade Pangolin nomenclature. JN.1* was the most common lineage (75.46%), followed by XBB.2.3* (8.92%) and XBB.1.16* (5.94%) (Table 1).

**Table 1:** Distribution of SARS-CoV-2 variants among sequences deposited on IBDC from India IBDC: Indian Biological Data Centre.

Clade	Nextclade Pangolin lineage		Count (%)	
21K	BA.1*	BA.1	2	23 (0.73%)
		BA.1.1	21	
21L	BA.2*	BA.2	31	56 (1.78%)
		BA.2.1	4	
		BA.2.10.4	1	
		BA.2.12	1	
		BA.2.38	13	
		BA.2.38.1	1	
		BA.2.76	1	
22C		BA.2.12.1	1	
		CH.1.1.24	1	
		DV.7.1.3	1	
		FK.1.1	1	
22B	BA.5*	BA.5	1	5 (0.16%)
		BA.5.5	1	
		BA.5.5.1	1	
		BE.1.1	1	
22E		BQ.1.22	1	
23A	XBB.1.5*	GF.1	1	11 (0.35%)
		JD.1.1	2	
		XBB.1.5	6	
		XBB.1.5.28	1	
23G		GK.1.1	1	
23D	XBB.1.9*	XBB.1.9.1	2	47 (1.49%)
		EG.5.2	1	
		FL.1.5	1	
		FL.1.5.1	2	
		FL.13.2	1	
		FL.13.4.1	1	
		FL.4.8	1	
		HN.5	1	
23F		EG.5.1	3	
		EG.5.1.1	5	
		EG.5.1.15	1	
		EG.5.1.3	1	
		EG.5.1.6	2	
		EG.5.1.8	1	
		HK.19	1	
		HK.29	1	
		HV.1	10	
		HV.1.11	2	
		JG.3	3	
		JG.3.2	1	
23H		HK.3	4	
		HK.3.1	1	
		HK.3.5	1	
23B	XBB.1.16*	XBB.1.16	57	187 (5.94%)
		XBB.1.16.1	17	
		XBB.1.16.11	37	
		XBB.1.16.12	3	
		XBB.1.16.13	1	
		XBB.1.16.17	32	
		XBB.1.16.18	3	
		XBB.1.16.2	1	
		XBB.1.16.24	28	
		XBB.1.16.8	1	
		FU.1	3	
		FU.3.1	1	
		JF.1.1	1	
		JM.2	2	
23E	XBB.2.3*	GE.1	85	281 (8.92%)
		GE.1.1	10	
		GJ.1	4	
		GJ.1.1	8	
		GJ.1.2	2	
		GJ.3	1	
		GJ.6	4	
		GS.1	1	
		GZ.1	5	
		HH.1	1	
		HH.2	5	
		HH.6	10	
		HH.8	1	
		JE.1	1	
		JY.1	44	
		JY.1.1	47	
		XBB.2.3	15	
		XBB.2.3.10	1	
		XBB.2.3.11	2	
		XBB.2.3.12	1	
		XBB.2.3.18	1	
		XBB.2.3.19	2	
		XBB.2.3.2	7	
		XBB.2.3.22	1	
		XBB.2.3.3	20	
		XBB.2.3.5	1	
		XBB.2.3.8	1	
23I	BA.2.86*	BA.2.86	15	32 (1.02%)
		BA.2.86.1	12	
		JN.2	3	
		JN.4	1	
		JN.6	1	
	JN.1*	JN.1	1026	2377 (75.46%)
		JN.1.1	959	
		JN.1.1.1	1	
		JN.1.1.3	1	
		JN.1.10	7	
		JN.1.11	304	
		JN.1.2	2	
		JN.1.3	2	
		JN.1.4	36	
		JN.1.5	21	
		JN.1.6	1	
		JN.1.7	1	
		JN.1.8	10	
		JN.1.9	6	
22F	Other XBBs	XBB	1	13 (0.41%)
		XBB.1	1	
		GW.5.1.1	3	
		GW.5.3.1	2	
		JC.2	2	
		KE.2	1	
		XBB.1.41.1	1	
		XBB.2.4	1	
		XBB.2.6	1	
Recombinant	Other recombinant variants	XAC	1	35 (1.11%)
		XAD	2	
		XAF	1	
		XAG	2	
		XAH	2	
		XAK	1	
		XBH	1	
		XBQ	1	
		XBW	1	
		XCG	1	
		XCH.1	1	
		XDA	12	
		XDA.1	1	
		XDB	3	
		XDD	4	
		XDK	1	
19A	Other lineages	B	4	44 (1.40%)
20A		B.1	7	
20B		B.1.1	11	
		B.1.1.161	4	
		B.1.1.220	1	
		B.1.1.519	2	
20C		B.1.566	1	
20F		D.2	1	
20G		B.1.2	2	
21C		B.1.429	3	
21J		AY.4	2	
21M		B.1.1.529	5	
22A		BA.4.6	1	
Lineage unassigned (due to poor quality sequences)			39 (1.24%)	
Grand total			3150	

**Figure 1:**
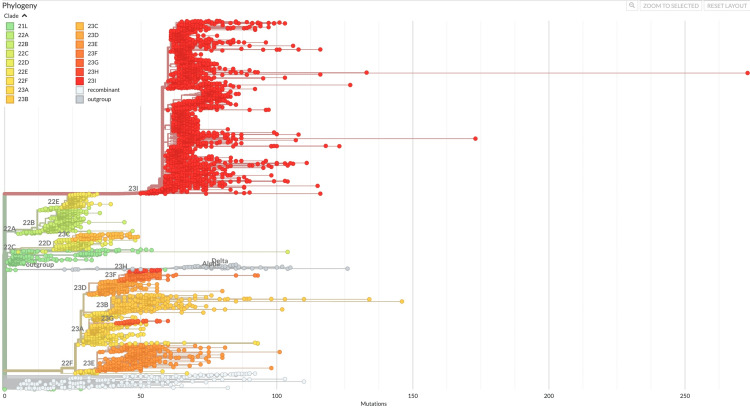
Evolutionary relationship of clade 23I with other clades in India The figure represents the phylogenetic relationship of clade 23I with other clades identified in Indian sequences during the study period. The horizontal axis of the phylogenetic tree denotes the number of mutations, with the numbers increasing progressively from left to right. The branches, represented by horizontal lines, signify evolutionary divergence over time. The length of the branch horizontally correlates with the amount of change. The vertical axis on the other hand organizes the clade labels in a spatial manner for clarity. It does not imply any phylogenetic or temporal relationship. Image credits: Rashmita Das

**Figure 2:**
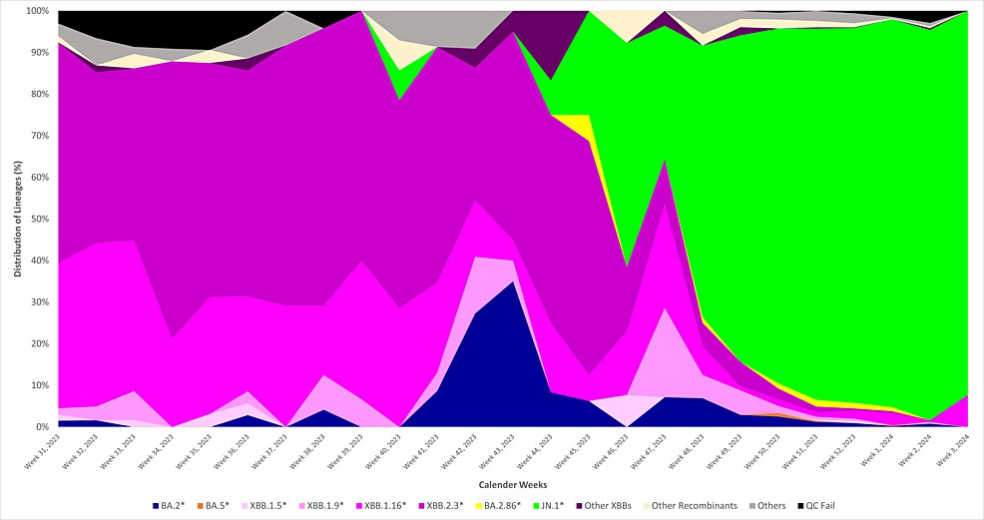
Temporal distribution of SARS-CoV-2 variants based on sequences submitted to the IBDC in India IBDC: Indian Biological Data Centre. Image credits: Rashmita Das

**Figure 3:**
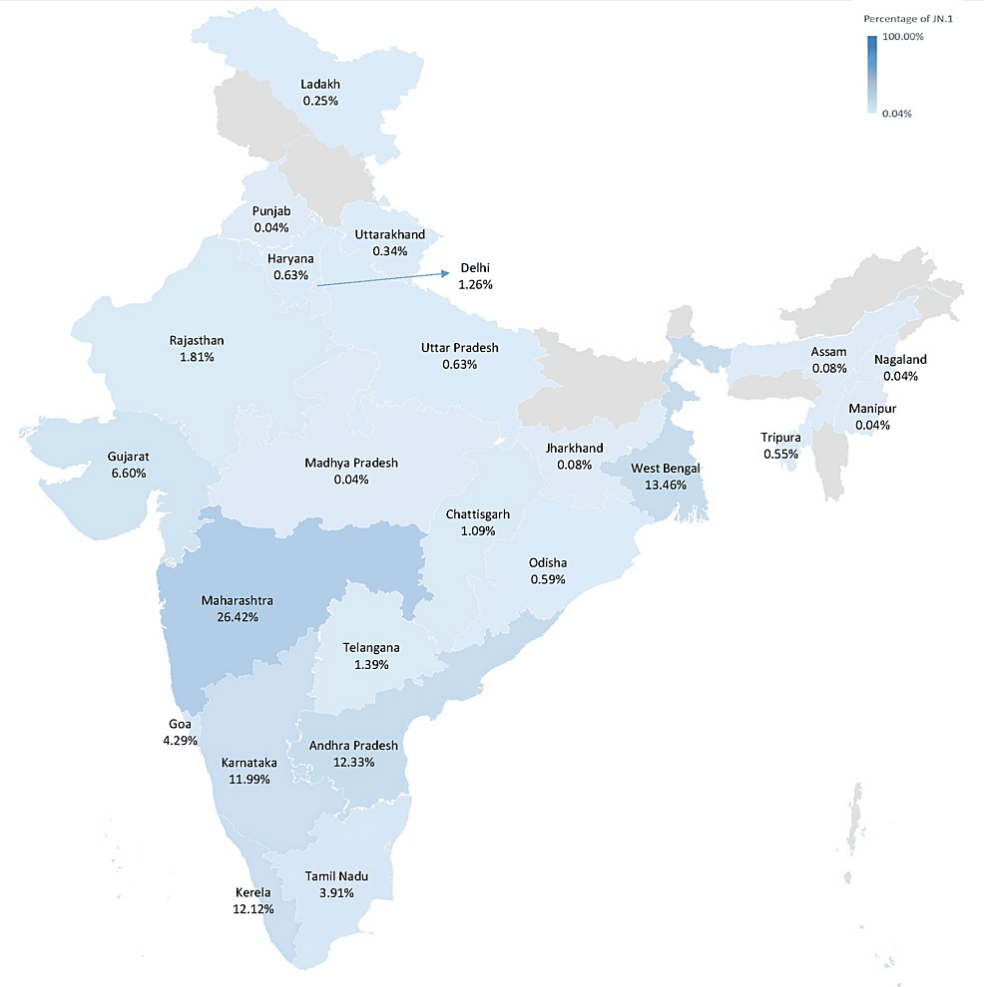
Geographic distribution of the 2377 JN.1* SARS-CoV-2 sequences based on sequences submitted to the IBDC in India IBDC: Indian Biological Data Centre. Image credits: Rashmita Das

Figure 1 illustrates the evolutionary relationship of the JN.1* variant (clade 23I) in comparison to other variants. In India, the temporal distribution of variants indicates that the XBB variant and its related lineages were predominant from week 31, 2023, until week 45, 2023 (Figure 2). The JN.1* variant was first identified in Indian sequences on 6th October 2023 in Kerala. Subsequently, by December 2023, the JN.1* variant became the most prevalent variant across India. The JN.1* variant has grown from 8.33% since its first detection in week 40, 2023, to 93.83% in week two, 2024. Of all the sequences submitted by various states, 75% are of the JN.1* variant, with the highest proportion of JN.1* sequences originating from Maharashtra (26.42%), followed by West Bengal (13.46%), Andhra Pradesh (12.33%), Kerala (12.12%), and Karnataka (11.99%) (Figure 3).

Test positivity and lineage distribution of SARS-CoV-2 in Maharashtra

**Figure 4:**
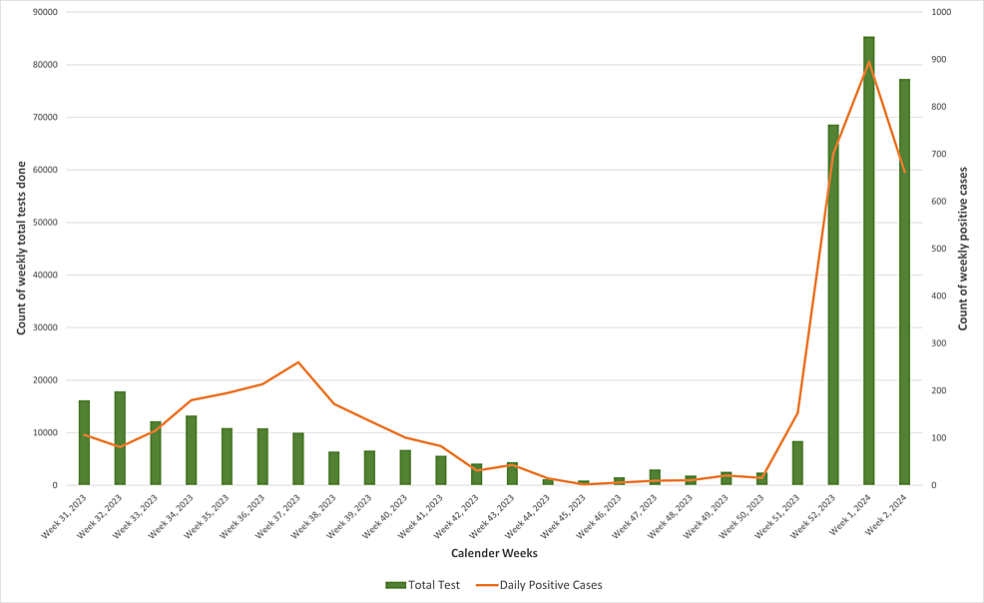
Weekly tests performed versus weekly count of positive cases in Maharashtra between 1st August 2023 and 15th January 2024 Image credits: Rashmita Das

Figure 4 represents the overview of SARS-CoV-2 testing in Maharashtra, tracking the total number of tests performed (including RT-PCR and antigen testing) and the positivity rate from the 31st week of 2023 through the second week of 2024. The total tests performed show significant variability with notable spikes toward the end of the period (in weeks 52 of 2023 and the first and second weeks of 2024), suggesting increased testing efforts during these weeks. Similarly, the weekly positivity rate also experienced fluctuations throughout the period. The highest positivity rate was 2.67% in the 38th week of 2023. Following this peak, the positivity rate gradually declined, reaching its lowest in the 45th week (0.21%) and increasing again to 1.81% in the 51st week. Following the first detection of the JN.1* variant globally, the testing efforts in the state and the country were increased [11], thus explaining the variations in both the number of tests performed and the positivity rate reflecting the changes in the testing strategies and the public health policies in the state.

**Figure 5:**
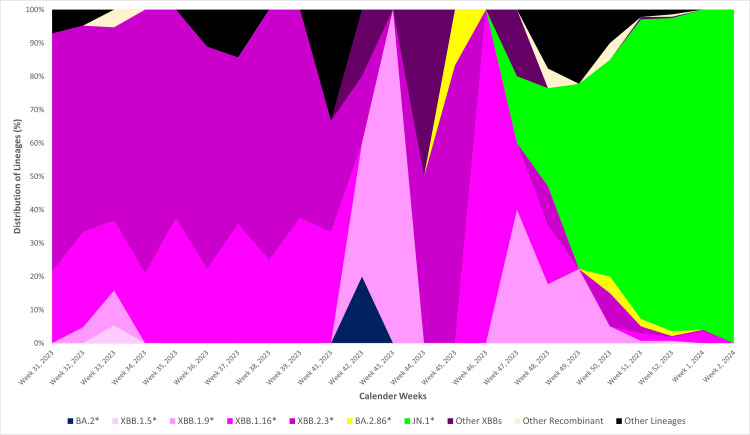
Temporal distribution of SARS-CoV-2 variants based on sequences submitted to the IBDC from Maharashtra IBDC: Indian Biological Data Centre. Image credits: Rashmita Das

Figure 5 presents the weekly distribution of SARS-CoV-2 variants in Maharashtra from week 31, 2023 to week two, 2024, mirroring the trend observed across India. The JN.1* variant was first identified on 23rd November 2023, and its prevalence rose from 20% in week 47, 2023, to 100% by the second week of 2024.

Demographic and clinical characteristics of JN.1* SARS-CoV-2 variant in Maharashtra

Out of 628 JN.1* cases in Maharashtra, 550 (87.58%) cases had complete metadata available and were included in the demographic study (Table 2). Of these 550 participants, 55.45% were males and 44.55% were females. The median age of the study population was 42 (IQR: 30-60) years, with individuals aged 60 years and above (25.09%) being predominantly affected.

**Table 2:** Demographic characteristics of JN.1* SARS-CoV-2 variant in Maharashtra (n = 550)

Demographic characteristics	Count (%)
Gender-wise distribution	
Male	305 (55.45%)
Female	245 (44.55%)
Age-wise distribution (in years)	
0-9	05 (0.91%)
10-19	39 (7.09%)
20-29	84 (15.27%)
30-39	115 (20.91%)
40-49	84 (15.27%)
50-59	85 (15.45%)
60 and above	138 (25.09%)
Area-wise distribution	
Ahmednagar	02 (0.36%)
Akola	02 (0.36%)
Amravati	18 (3.27%)
Chhatrapati Sambhajinagar	54 (9.82%)
Beed	03 (0.55%)
Bhandara	01 (0.18%)
Hingoli	03 (0.55%)
Jalgaon	04 (0.73%)
Jalna	02 (0.36%)
Kolhapur	10 (1.82%)
Mumbai	18 (3.27%)
Nagpur	36 (6.55%)
Nanded	02 (0.36%)
Nandurbar	01 (0.18%)
Nashik	02 (0.36%)
Osmanabad	02 (0.36%)
Pune	273 (49.64%)
Raigad	18 (3.27%)
Sangli	01 (0.18%)
Satara	01 (0.18%)
Solapur	13 (2.36%)
Thane	83 (15.09%)
Yavatmal	01 (0.18%)

From this subset of 550 cases, attempts to contact 253 (46%) participants were unsuccessful due to non-response to calls, and 18 (3.27%) participants declined participation in the study. Therefore, 279 (50.73%) participants were successfully contacted and included in the clinical study. Table 3 summarizes the clinical characteristics, vaccination status, and outcome of JN.1* cases in Maharashtra. Most JN.1* cases had symptomatic disease (95.34%) with mild symptoms. The most common symptoms were cold (67.03%), fever (55.91%), cough (40.86%), headache (15.64%), body ache (9.68%), and fatigue (8.60%). Underlying comorbid conditions, either alone or in combination, were reported in 17.56% (49 out of 279) of cases, with diabetes mellitus (29, alone or in combination; 59.18%), hypertension (22, alone or in combination; 44.90%), and underlying lung pathology (six, alone or in combination; 12.25%) being the most common conditions.

**Table 3:** Clinical characteristics of JN.1* SARS-CoV-2 variant in Maharashtra (n = 279) Out of 550 JN.1* cases with complete metadata, 279 participants were successfully contacted and included in the clinical study.

Clinical characteristics	Count (%)
1. History of previous infection	
Present	41 (14.69%)
Absent	238 (85.31%)
2. Presence of comorbidities	
a. Present	49 (17.56%)
i. Diabetes mellitus (DM)	18 (36.73%)
ii. Hypertension (HTN)	12 (24.29%)
iii. Underlying lung disease	04 (8.16%)
iv. Arthritis	01 (2.04%)
v. Tuberculosis	01 (2.04%)
vi. Hypothyroidism	01 (2.04%)
vii. Coronary heart disease (CHD)	01 (2.04%)
viii. HIV/AIDS	01 (2.04%)
ix. DM + HTN	08 (16.33%)
x. DM + underlying lung disease	01 (2.04%)
xi. DM + HTN + CHD	01 (2.04%)
xii. DM + HTN + underlying lung disease	01 (2.04%)
b. Absent	230 (82.44%)
3. Vaccination status	
a. Vaccinated	263 (94.26%)
i. One dose	12 (4.56%)
ii. Two doses	151 (57.42%)
iii. Booster dose (precautionary dose)	100 (38.02%)
b. Not vaccinated	14 (5.02%)
c. Data not available	02 (0.72%)
4. Symptom status	
a. Symptomatic	266 (95.34%)
b. Asymptomatic	13 (4.66%)
5. Presenting symptoms	
a. Fever	156 (55.91%)
b. Cold	187 (67.03%)
c. Cough	114 (40.86%)
d. Breathlessness	15 (5.38%)
e. Headache	28 (15.64%)
f. Fatigue	24 (8.60%)
g. Body ache	27 (9.68%)
h. Diarrhea	02 (0.72%)
i. Sore throat	16 (5.74%)
j. Vomiting	02 (0.72%)
k. Chest pain	01 (0.36%)
l. Loss of taste and smell	01 (0.36%)
m. Joint pain	01 (0.36%)
6. Type of quarantine	
a. Home isolation	242 (86.74%)
b. Hospital isolation/hospitalization	37 (13.26%)
7. Treatment	
a. Received no treatment	03 (1.075%)
b. Received symptomatic treatment	259 (92.83%)
c. Received oxygen therapy	14 (5.02%)
i. Non-invasive ventilation	09 (64.29%)
ii. Invasive ventilation	05 (35.71%)
d. Received antiviral/steroid with oxygen	03 (1.075%)
8. Outcome	
a. Alive	275 (98.57%)
b. Dead	04 (1.43%)

Regarding the vaccination status, 94.26% (263 out of 279) of cases received at least one dose of the COVID-19 vaccine, while 5.02% (14 out of 279) were not vaccinated. Most unvaccinated individuals were children aged zero to nine years (five out of 14, 35.71%) and adults aged 30-39 years (three out of 14, 21.43%). Covishield (ChAdOx1nCoV-19 coronavirus vaccine) (242 out of 263, 92.01%) was the predominant vaccine administered, followed by Covaxin^TM^ (BBV152A-a whole inactivated virus-based COVID-19 vaccine) (18 out of 263, 6.84%).

Of all the cases, 13.26% (37 out of 279) required institutional quarantine or hospitalization. Nearly all of the patients who were home-isolated received conservative treatment (240 out of 242, 99.17%), whereas hospitalized patients often received conservative care (20 out of 37, 54.05%) or required supplemental oxygen therapy (17 out of 37, 45.95%). The recovery rate was high; out of 279 cases, 98.57% of cases (275 out of 279) recovered from the disease, while 1.43% (four out of 279) succumbed.

Among the deceased, three were over 60 years of age (75%), and one (25%) was in the age group of 30-39 years. Three out of four cases (75%) suffered from respiratory symptoms and had underlying comorbidities, while one (25%) did not have respiratory symptoms and was admitted to the hospital due to paralysis. Of the three cases with respiratory symptoms, two were aged over 60 years with pre-existing conditions such as chronic obstructive pulmonary disease in one, and diabetes mellitus and hypertension in the other. The third was an immunocompromised individual with HIV/AIDS. Regarding vaccination, two were vaccinated with two doses, one received three doses of vaccine, and the vaccination status was unclear in one.

Weekly trends in isolation bed occupancy in hospitals, oxygen/ventilator support, and COVID-19-positive cases in Maharashtra

**Figure 6:**
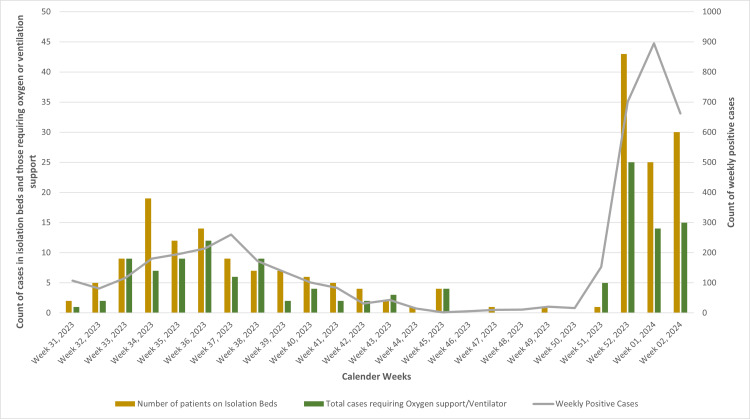
Weekly trends in isolation bed occupancy in hospitals, oxygen/ventilator support, and COVID-19-positive cases in Maharashtra Image credits: Rashmita Das

Out of the total positive cases in the state, 8.03% (338 out of 4211) required hospital admission. Among these hospitalized cases, 61.24% (207 out of 338) were managed in isolation beds without the need for oxygen support, while the remaining 38.76% (131 out of 338) needed oxygen or ventilator support. Mirroring the state's positivity rate trend, the patterns of hospital admissions and the need for oxygen support also showed fluctuations during the same timeframe (Figure 6).

## Discussion

Although the BA.2.86 variant was first reported in August 2023 globally, it did not exhibit significant growth advantages or abilities to evade the humoral immune response compared to the global dominant variants such as EG.5.1 and HK.3 [12]. Over four weeks, from week 40 to week 44 of 2023, the percentage of BA.2.86 sequences slowly and steadily increased from 1.8% to 8.9% [4]. Conversely, the JN.1 variant, distinguished by a single additional mutation (L455S) in its spike protein compared to BA.2.86, swiftly ascended to global dominance. Within the same duration of four weeks, from week 44 to week 48 of 2023, its global prevalence surged from 3.3% to 27.1% [13], making up 95% of all sequences collected globally in February 2024 [14]. The quick shift from XBB sub-lineages to JN.1* mirrors the transition of Delta to the Omicron variant. In the Omicron wave, Omicron's prevalence increased from under 0.1% to 89.1%, making it the globally dominant variant within nine weeks [15,16]. The rapid emergence and dominance of the JN.1 variant in India and globally can be attributed to its enhanced transmissibility due to spike mutation L455S. In the early stages of its emergence, analysis of JN.1's effective reproductive number (Ro) using sequences from France, the United Kingdom, and Spain indicated that the JN.1 Ro exceeded that of both BA.2.86.1 and HK.3, with the latter being among the XBB lineages with the most significant growth advantage as of late November 2023. This analysis predicted JN.1's trajectory toward global dominance, a status that it had achieved in France and Spain by the close of November 2023 [9]. Presently, the JN.1* variant exhibits a relative growth advantage of 57% (confidence interval: 56-57%) [17].

The in vitro angiotensin-converting enzyme 2 (ACE2)-binding assay [9] and surface plasmon resonance analysis [12] using the RBD (monomeric) of JN.1 and BA.2.86 showed a significant reduction in the affinity of the JN.1 RBD when compared to the BA.2.86 RBD for human ACE2 receptor. Conversely, the pseudovirus assay using trimerized whole spike protein showed that the infectivity rate of JN.1 was substantially greater than that of BA.2.86 [9]. The neutralization assays demonstrated a superior ability of the JN.1 variant to evade the immune response compared to BA.2.86, with a 2.1-fold decrease in neutralization titers against antibodies from individuals reinfected with XBB after BA.5/BF.7 infection, and a 1.1-fold reduction from those with XBB breakthrough infections [9,12]. It was also shown that a small change in the L455, which is predominantly located at the epitope of RBD class 1 antibodies, enhanced JN.1's ability to evade class 1 monoclonal antibodies, making up for the fact that the parent variant, BA.2.86, was susceptible to this antibody group. However, one of the therapeutic antibodies, SA55, was shown to retain its efficacy against JN.1 [12]. As indicated by the pseudovirus assay and neutralization assays mentioned earlier, JN.1 has increased infectivity and immune evasion capabilities compared to BA.2.86.

The JN.1 variant caused mild infections in Maharashtra, mirroring the clinical outcomes observed during the previous waves in the state caused by BA.2.75, XBB, and XBB.1.16, with similar rates of hospital admissions, oxygen requirements, and mortality. In contrast to earlier waves where individuals aged 21-40 years were primarily affected, the current wave has seen a significant shift, with those aged 60 years and above being predominantly affected [18-20]. Despite a notable increase in number of positive cases, health services data showed that the overall hospitalization rate during the study period was 8.03%, and oxygen support was needed in 3.11% of positive cases, reiterating our observations that the uptick in case positivity did not significantly impact the hospitalization or oxygen demand in the state.

This evolutionary pattern of JN.1 arising from BA.2.86 is similar to the previous transition of CH.1.1 (BA.2.75.3.4.1.1.1.1) arising from BA.2.75 [12]. Albeit the addition of a new mutation in JN.1 helped it to overcome the competition faced by the parent BA.2.86 from the existing dominant XBB variants. On the other hand, mutations of CH.1.1 helped to evade the neutralizing antibodies developed in the hosts against the parent lineage, i.e., BA.2.75, which had caused widespread infection in the community. The emergence of XBB due to recombination of BJ.1 (BA.2.10.1) and BM.1.1.1 (BA.2.75.3.1.1.1) also demonstrates the innovative evolutionary strategy of SARS-CoV-2 to evade the existing neutralizing antibodies against the parent lineages. This highlights the importance of closely monitoring variants with high ACE2-binding affinity and distinct antigenic characteristics. Hence, the early detection of these mutations is crucial before they lead to evident health implications, a task that case-based surveillance might not adequately accomplish. Instead, implementing broad, population-level surveillance methods, like wastewater-based surveillance, is essential for uncovering cryptic mutations and variants. For instance, a study in Berlin, Germany, demonstrated the effectiveness of this approach by identifying the JN.1 variant in wastewater three weeks before it was detected in clinical samples, highlighting the value of population-based surveillance in early detection efforts [21]. Therefore, prompt identification of novel SARS-CoV-2 variants is vital for facilitating rapid risk evaluations, timely dissemination of information, and coordinated public health responses.

The study suffers from a few limitations. First, the analysis depends on individuals who tested for COVID-19, allowing their samples to be sequenced, and individuals who responded and consented to the study, suggesting that the reported figures might not represent the actual burden of the disease. Secondly, the study is region-specific, with data from Maharashtra. Therefore, the findings of this study might restrict its generalizability to other geographic areas and populations with different demographic characteristics. Additionally, the clinical data were obtained through telephonic conversation, potentially leading to recall bias among patients. Therefore, more comprehensive studies are needed to enhance representativeness and accuracy, including studies in diverse geographic locations and populations and using a wider range of data collection methods.

## Conclusions

The JN.1* variant has emerged as the predominant SARS-CoV-2 variant in India, with clinical presentation similar to previous variants circulating in Maharashtra, India. Notably, while JN.1* has not been associated with more severe disease outcomes, its enhanced transmissibility underscores the importance of studying the ongoing viral evolution. The evolution of the virus by adding novel mutations necessitates prompt detection strategies to prevent potential surges. The pressing necessity for swift identification and the clinical features of new variants will enable public health authorities to predict and mitigate the risks effectively, ensuring preparedness against future outbreaks.

## Appendices

**Table 4:** IDs of the sequences used in the study, downloaded from the Indian Biological Data Centre (IBDC), Regional Centre for Biotechnology, Department of Biotechnology, Ministry of Science and Technology, Government of India, with their kind permission

IDs of the sequences used in the study, downloaded from the Indian Biological Data Centre (IBDC), Regional Centre for Biotechnology, Department of Biotechnology, Ministry of Science and Technology, Government of India, with their kind permission
INCOV286977
INCOV286992
INCOV287059
INCOV287062
INCOV287064
INCOV287066
INCOV287067
INCOV287068
INCOV287069
INCOV287083
INCOV287086
INCOV287087
INCOV287106
INCOV287107
INCOV287108
INCOV287109
INCOV287110
INCOV287111
INCOV287112
INCOV287113
INCOV287117
INCOV287118
INCOV287119
INCOV287121
INCOV287123
INCOV287124
INCOV287125
INCOV287126
INCOV287127
INCOV287129
INCOV287137
INCOV287138
INCOV287140
INCOV287141
INCOV287146
INCOV287147
INCOV287167
INCOV287168
INCOV287169
INCOV287170
INCOV287171
INCOV287172
INCOV287173
INCOV287174
INCOV287175
INCOV287176
INCOV287177
INCOV287178
INCOV287179
INCOV287180
INCOV287181
INCOV287182
INCOV287183
INCOV287184
INCOV287186
INCOV287187
INCOV287188
INCOV287189
INCOV287190
INCOV287191
INCOV287192
INCOV287193
INCOV287194
INCOV287195
INCOV287196
INCOV287197
INCOV287198
INCOV287199
INCOV287200
INCOV287201
INCOV287202
INCOV287203
INCOV287204
INCOV287205
INCOV287206
INCOV287207
INCOV287208
INCOV287209
INCOV287210
INCOV287211
INCOV287212
INCOV287213
INCOV287214
INCOV287215
INCOV287216
INCOV287217
INCOV287218
INCOV287219
INCOV287220
INCOV287221
INCOV287222
INCOV287223
INCOV287224
INCOV287225
INCOV287226
INCOV287227
INCOV287228
INCOV287229
INCOV287230
INCOV287231
INCOV287232
INCOV287233
INCOV287234
INCOV287235
INCOV287236
INCOV287237
INCOV287238
INCOV287239
INCOV287240
INCOV287241
INCOV287242
INCOV287243
INCOV287244
INCOV287245
INCOV287246
INCOV287247
INCOV287248
INCOV287249
INCOV287250
INCOV287251
INCOV287252
INCOV287253
INCOV287257
INCOV287258
INCOV287265
INCOV287266
INCOV287267
INCOV287268
INCOV287269
INCOV287270
INCOV287271
INCOV287272
INCOV287273
INCOV287274
INCOV287275
INCOV287277
INCOV287278
INCOV287279
INCOV287280
INCOV287281
INCOV287282
INCOV287283
INCOV287284
INCOV287286
INCOV287287
INCOV287288
INCOV287289
INCOV287293
INCOV287294
INCOV287295
INCOV287296
INCOV287297
INCOV287298
INCOV287299
INCOV287300
INCOV287302
INCOV287308
INCOV287309
INCOV287310
INCOV287311
INCOV287312
INCOV287313
INCOV287372
INCOV287373
INCOV287374
INCOV287375
INCOV287376
INCOV287377
INCOV287378
INCOV287379
INCOV287380
INCOV287381
INCOV287382
INCOV287383
INCOV287384
INCOV287385
INCOV287386
INCOV287387
INCOV287388
INCOV287389
INCOV287390
INCOV287391
INCOV287392
INCOV287393
INCOV287394
INCOV287395
INCOV287396
INCOV287397
INCOV287398
INCOV287399
INCOV287400
INCOV287401
INCOV287402
INCOV287403
INCOV287404
INCOV287554
INCOV287555
INCOV287556
INCOV287558
INCOV287559
INCOV287560
INCOV287561
INCOV287562
INCOV287567
INCOV287568
INCOV287569
INCOV287570
INCOV287571
INCOV287572
INCOV287573
INCOV287574
INCOV287575
INCOV287576
INCOV287577
INCOV287578
INCOV287579
INCOV287580
INCOV287581
INCOV287585
INCOV287587
INCOV287588
INCOV287589
INCOV287590
INCOV287591
INCOV287592
INCOV287593
INCOV287893
INCOV287894
INCOV287895
INCOV287896
INCOV287897
INCOV287898
INCOV287899
INCOV287900
INCOV287901
INCOV287902
INCOV287903
INCOV287904
INCOV287905
INCOV287906
INCOV287907
INCOV287908
INCOV287909
INCOV287910
INCOV287950
INCOV287951
INCOV287952
INCOV287953
INCOV287954
INCOV287955
INCOV287956
INCOV287957
INCOV287958
INCOV287959
INCOV287961
INCOV287962
INCOV287963
INCOV287964
INCOV287965
INCOV287967
INCOV287968
INCOV287969
INCOV287970
INCOV287971
INCOV287972
INCOV287973
INCOV287974
INCOV287975
INCOV287976
INCOV287977
INCOV287978
INCOV287979
INCOV287980
INCOV287981
INCOV287982
INCOV287983
INCOV287984
INCOV287985
INCOV287986
INCOV287987
INCOV287988
INCOV287989
INCOV287990
INCOV287991
INCOV287992
INCOV287993
INCOV287994
INCOV287995
INCOV287996
INCOV287997
INCOV287998
INCOV287999
INCOV288000
INCOV288001
INCOV288002
INCOV288003
INCOV288004
INCOV288005
INCOV288006
INCOV288007
INCOV288008
INCOV288009
INCOV288012
INCOV288013
INCOV288015
INCOV288016
INCOV288017
INCOV288018
INCOV288019
INCOV288020
INCOV288021
INCOV288022
INCOV288023
INCOV288024
INCOV288025
INCOV288026
INCOV288029
INCOV288030
INCOV288031
INCOV288033
INCOV288034
INCOV288035
INCOV288036
INCOV288037
INCOV288038
INCOV288039
INCOV288040
INCOV288041
INCOV288042
INCOV288043
INCOV288045
INCOV288046
INCOV288047
INCOV288048
INCOV288049
INCOV288050
INCOV288051
INCOV288052
INCOV288053
INCOV288054
INCOV288055
INCOV288056
INCOV288057
INCOV288058
INCOV288059
INCOV288060
INCOV288061
INCOV288062
INCOV288063
INCOV288064
INCOV288065
INCOV288066
INCOV288067
INCOV288068
INCOV288069
INCOV288070
INCOV288071
INCOV288072
INCOV288073
INCOV288074
INCOV288075
INCOV288076
INCOV288077
INCOV288078
INCOV288079
INCOV288080
INCOV288081
INCOV288083
INCOV288084
INCOV288085
INCOV288087
INCOV288088
INCOV288089
INCOV288095
INCOV288096
INCOV288098
INCOV288099
INCOV288114
INCOV288116
INCOV288117
INCOV288120
INCOV288121
INCOV288122
INCOV288123
INCOV288124
INCOV288125
INCOV288126
INCOV288127
INCOV288128
INCOV288129
INCOV288130
INCOV288131
INCOV288132
INCOV288133
INCOV288134
INCOV288135
INCOV288136
INCOV288137
INCOV288138
INCOV288139
INCOV288140
INCOV288141
INCOV288142
INCOV288143
INCOV288144
INCOV288145
INCOV288146
INCOV288147
INCOV288148
INCOV288149
INCOV288150
INCOV288151
INCOV288152
INCOV288153
INCOV288154
INCOV288155
INCOV288156
INCOV288170
INCOV288171
INCOV288172
INCOV288173
INCOV288174
INCOV288175
INCOV288176
INCOV288177
INCOV288178
INCOV288179
INCOV288180
INCOV288181
INCOV288182
INCOV288183
INCOV288184
INCOV288185
INCOV288186
INCOV288207
INCOV288208
INCOV288209
INCOV288210
INCOV288211
INCOV288212
INCOV288213
INCOV288214
INCOV288215
INCOV288216
INCOV288217
INCOV288218
INCOV288219
INCOV288220
INCOV288221
INCOV288222
INCOV288223
INCOV288224
INCOV288225
INCOV288226
INCOV288227
INCOV288228
INCOV288229
INCOV288230
INCOV288231
INCOV288232
INCOV288233
INCOV288234
INCOV288235
INCOV288236
INCOV288237
INCOV288238
INCOV288239
INCOV288240
INCOV288241
INCOV288242
INCOV288243
INCOV288244
INCOV288246
INCOV288247
INCOV288248
INCOV288249
INCOV288250
INCOV288251
INCOV288252
INCOV288253
INCOV288254
INCOV288255
INCOV288256
INCOV288257
INCOV288258
INCOV288259
INCOV288260
INCOV288261
INCOV288262
INCOV288263
INCOV288264
INCOV288265
INCOV288266
INCOV288267
INCOV288268
INCOV288269
INCOV288270
INCOV288271
INCOV288272
INCOV288273
INCOV288274
INCOV288275
INCOV288276
INCOV288277
INCOV288278
INCOV288279
INCOV288280
INCOV288281
INCOV288282
INCOV288283
INCOV288284
INCOV288285
INCOV288286
INCOV288287
INCOV288288
INCOV288289
INCOV288290
INCOV288291
INCOV288292
INCOV288293
INCOV288294
INCOV288295
INCOV288296
INCOV288297
INCOV288298
INCOV288299
INCOV288300
INCOV288301
INCOV288302
INCOV288303
INCOV288304
INCOV288305
INCOV288306
INCOV288307
INCOV288308
INCOV288309
INCOV288310
INCOV288311
INCOV288312
INCOV288313
INCOV288314
INCOV288315
INCOV288316
INCOV288317
INCOV288318
INCOV288319
INCOV288320
INCOV288321
INCOV288322
INCOV288323
INCOV288324
INCOV288325
INCOV288326
INCOV288327
INCOV288328
INCOV288329
INCOV288330
INCOV288331
INCOV288332
INCOV288333
INCOV288334
INCOV288335
INCOV288336
INCOV288337
INCOV288338
INCOV288339
INCOV288340
INCOV288341
INCOV288342
INCOV288343
INCOV288344
INCOV288345
INCOV288346
INCOV288347
INCOV288348
INCOV288349
INCOV288350
INCOV288351
INCOV288352
INCOV288353
INCOV288354
INCOV288355
INCOV288356
INCOV288357
INCOV288358
INCOV288359
INCOV288360
INCOV288361
INCOV288362
INCOV288363
INCOV288364
INCOV288365
INCOV288366
INCOV288367
INCOV288368
INCOV288369
INCOV288370
INCOV288371
INCOV288372
INCOV288373
INCOV288374
INCOV288375
INCOV288376
INCOV288377
INCOV288378
INCOV288379
INCOV288380
INCOV288381
INCOV288382
INCOV288383
INCOV288384
INCOV288385
INCOV288386
INCOV288387
INCOV288388
INCOV288389
INCOV288390
INCOV288391
INCOV288392
INCOV288393
INCOV288394
INCOV288395
INCOV288396
INCOV288397
INCOV288398
INCOV288399
INCOV288400
INCOV288401
INCOV288402
INCOV288403
INCOV288404
INCOV288405
INCOV288406
INCOV288407
INCOV288408
INCOV288409
INCOV288410
INCOV288411
INCOV288412
INCOV288413
INCOV288414
INCOV288415
INCOV288416
INCOV288417
INCOV288418
INCOV288419
INCOV288420
INCOV288421
INCOV288422
INCOV288423
INCOV288424
INCOV288425
INCOV288426
INCOV288427
INCOV288428
INCOV288429
INCOV288430
INCOV288431
INCOV288432
INCOV288433
INCOV288434
INCOV288435
INCOV288436
INCOV288437
INCOV288438
INCOV288439
INCOV288440
INCOV288441
INCOV288442
INCOV288443
INCOV288444
INCOV288445
INCOV288446
INCOV288447
INCOV288448
INCOV288449
INCOV288450
INCOV288451
INCOV288452
INCOV288453
INCOV288454
INCOV288455
INCOV288456
INCOV288457
INCOV288458
INCOV288459
INCOV288460
INCOV288461
INCOV288462
INCOV288463
INCOV288464
INCOV288465
INCOV288466
INCOV288467
INCOV288468
INCOV288469
INCOV288470
INCOV288471
INCOV288472
INCOV288473
INCOV288474
INCOV288475
INCOV288476
INCOV288477
INCOV288478
INCOV288479
INCOV288480
INCOV288481
INCOV288482
INCOV288483
INCOV288484
INCOV288485
INCOV288486
INCOV288487
INCOV288488
INCOV288489
INCOV288490
INCOV288491
INCOV288492
INCOV288493
INCOV288494
INCOV288495
INCOV288496
INCOV288497
INCOV288498
INCOV288499
INCOV288500
INCOV288501
INCOV288502
INCOV288503
INCOV288504
INCOV288505
INCOV288506
INCOV288507
INCOV288508
INCOV288509
INCOV288510
INCOV288511
INCOV288512
INCOV288513
INCOV288514
INCOV288515
INCOV288516
INCOV288517
INCOV288518
INCOV288519
INCOV288520
INCOV288521
INCOV288522
INCOV288523
INCOV288524
INCOV288525
INCOV288526
INCOV288527
INCOV288528
INCOV288529
INCOV288530
INCOV288531
INCOV288532
INCOV288533
INCOV288534
INCOV288535
INCOV288536
INCOV288537
INCOV288538
INCOV288539
INCOV288540
INCOV288541
INCOV288542
INCOV288543
INCOV288544
INCOV288545
INCOV288546
INCOV288547
INCOV288548
INCOV288549
INCOV288550
INCOV288551
INCOV288552
INCOV288553
INCOV288554
INCOV288555
INCOV288556
INCOV288557
INCOV288558
INCOV288559
INCOV288560
INCOV288561
INCOV288562
INCOV288563
INCOV288564
INCOV288567
INCOV288568
INCOV288569
INCOV288570
INCOV288571
INCOV288572
INCOV288573
INCOV288574
INCOV288575
INCOV288576
INCOV288577
INCOV288578
INCOV288579
INCOV288580
INCOV288581
INCOV288582
INCOV288583
INCOV288584
INCOV288585
INCOV288586
INCOV288587
INCOV288588
INCOV288589
INCOV288590
INCOV288591
INCOV288592
INCOV288593
INCOV288594
INCOV288595
INCOV288596
INCOV288597
INCOV288598
INCOV288599
INCOV288600
INCOV288601
INCOV288602
INCOV288603
INCOV288604
INCOV288605
INCOV288606
INCOV288607
INCOV288608
INCOV288609
INCOV288610
INCOV288611
INCOV288612
INCOV288613
INCOV288614
INCOV288615
INCOV288616
INCOV288617
INCOV288618
INCOV288619
INCOV288620
INCOV288621
INCOV288622
INCOV288623
INCOV288624
INCOV288625
INCOV288626
INCOV288627
INCOV288628
INCOV288629
INCOV288630
INCOV288631
INCOV288632
INCOV288633
INCOV288634
INCOV288635
INCOV288636
INCOV288637
INCOV288638
INCOV288639
INCOV288640
INCOV288641
INCOV288642
INCOV288643
INCOV288644
INCOV288645
INCOV288646
INCOV288647
INCOV288648
INCOV288649
INCOV288650
INCOV288651
INCOV288652
INCOV288653
INCOV288654
INCOV288655
INCOV288656
INCOV288657
INCOV288658
INCOV288659
INCOV288660
INCOV288661
INCOV288662
INCOV288663
INCOV288664
INCOV288665
INCOV288666
INCOV288667
INCOV288668
INCOV288669
INCOV288670
INCOV288671
INCOV288672
INCOV288673
INCOV288674
INCOV288675
INCOV288676
INCOV288677
INCOV288678
INCOV288679
INCOV288680
INCOV288681
INCOV288682
INCOV288683
INCOV288684
INCOV288685
INCOV288686
INCOV288687
INCOV288688
INCOV288689
INCOV288690
INCOV288691
INCOV288692
INCOV288693
INCOV288694
INCOV288695
INCOV288696
INCOV288697
INCOV288698
INCOV288699
INCOV288700
INCOV288701
INCOV288702
INCOV288703
INCOV288704
INCOV288705
INCOV288706
INCOV288707
INCOV288708
INCOV288709
INCOV288710
INCOV288711
INCOV288712
INCOV288713
INCOV288714
INCOV288715
INCOV288716
INCOV288717
INCOV288718
INCOV288719
INCOV288720
INCOV288721
INCOV288722
INCOV288723
INCOV288724
INCOV288725
INCOV288726
INCOV288727
INCOV288728
INCOV288729
INCOV288730
INCOV288731
INCOV288732
INCOV288733
INCOV288734
INCOV288735
INCOV288736
INCOV288737
INCOV288738
INCOV288739
INCOV288740
INCOV288741
INCOV288742
INCOV288743
INCOV288744
INCOV288745
INCOV288746
INCOV288747
INCOV288748
INCOV288749
INCOV288750
INCOV288751
INCOV288752
INCOV288753
INCOV288754
INCOV288755
INCOV288756
INCOV288757
INCOV288758
INCOV288759
INCOV288760
INCOV288761
INCOV288762
INCOV288763
INCOV288764
INCOV288765
INCOV288766
INCOV288767
INCOV288768
INCOV288769
INCOV288770
INCOV288771
INCOV288773
INCOV288774
INCOV288775
INCOV288776
INCOV288777
INCOV288778
INCOV288779
INCOV288780
INCOV288781
INCOV288782
INCOV288783
INCOV288784
INCOV288785
INCOV288786
INCOV288787
INCOV288788
INCOV288789
INCOV288790
INCOV288791
INCOV288792
INCOV288793
INCOV288794
INCOV288795
INCOV288796
INCOV288797
INCOV288798
INCOV288799
INCOV288800
INCOV288801
INCOV288802
INCOV288803
INCOV288804
INCOV288805
INCOV288806
INCOV288807
INCOV288808
INCOV288809
INCOV288810
INCOV288811
INCOV288812
INCOV288813
INCOV288814
INCOV288815
INCOV288816
INCOV288817
INCOV288818
INCOV288819
INCOV288820
INCOV288821
INCOV288822
INCOV288823
INCOV288824
INCOV288825
INCOV288826
INCOV288827
INCOV288828
INCOV288829
INCOV288830
INCOV288831
INCOV288832
INCOV288833
INCOV288834
INCOV288835
INCOV288836
INCOV288837
INCOV288838
INCOV288839
INCOV288840
INCOV288841
INCOV288842
INCOV288843
INCOV288844
INCOV288845
INCOV288846
INCOV288847
INCOV288848
INCOV288849
INCOV288850
INCOV288851
INCOV288852
INCOV288853
INCOV288854
INCOV288855
INCOV288856
INCOV288857
INCOV288860
INCOV288861
INCOV288862
INCOV288863
INCOV288864
INCOV288865
INCOV288866
INCOV288867
INCOV288868
INCOV288869
INCOV288870
INCOV288871
INCOV288872
INCOV288873
INCOV288874
INCOV288875
INCOV288876
INCOV288877
INCOV288878
INCOV288879
INCOV288880
INCOV288881
INCOV288882
INCOV288883
INCOV288884
INCOV288885
INCOV288886
INCOV288887
INCOV288888
INCOV288889
INCOV288890
INCOV288891
INCOV288892
INCOV288893
INCOV288894
INCOV288895
INCOV288896
INCOV288897
INCOV288898
INCOV288899
INCOV288900
INCOV288901
INCOV288902
INCOV288903
INCOV289049
INCOV289050
INCOV289051
INCOV289053
INCOV289054
INCOV289055
INCOV289056
INCOV289057
INCOV289058
INCOV289059
INCOV289060
INCOV289061
INCOV289062
INCOV289063
INCOV289064
INCOV289065
INCOV289066
INCOV289067
INCOV289068
INCOV289069
INCOV289070
INCOV289071
INCOV289072
INCOV289073
INCOV289074
INCOV289075
INCOV289076
INCOV289077
INCOV289078
INCOV289079
INCOV289080
INCOV289081
INCOV289082
INCOV289083
INCOV289084
INCOV289085
INCOV289086
INCOV289087
INCOV289088
INCOV289089
INCOV289090
INCOV289091
INCOV289092
INCOV289093
INCOV289094
INCOV289095
INCOV289096
INCOV289097
INCOV289098
INCOV289099
INCOV289100
INCOV289101
INCOV289102
INCOV289103
INCOV289104
INCOV289105
INCOV289106
INCOV289107
INCOV289108
INCOV289109
INCOV289110
INCOV289111
INCOV289112
INCOV289113
INCOV289114
INCOV289115
INCOV289116
INCOV289117
INCOV289118
INCOV289119
INCOV289120
INCOV289121
INCOV289122
INCOV289123
INCOV289124
INCOV289125
INCOV289126
INCOV289127
INCOV289128
INCOV289129
INCOV289130
INCOV289131
INCOV289132
INCOV289133
INCOV289134
INCOV289135
INCOV289136
INCOV289137
INCOV289138
INCOV289139
INCOV289140
INCOV289141
INCOV289142
INCOV289143
INCOV289144
INCOV289145
INCOV289146
INCOV289147
INCOV289148
INCOV289149
INCOV289150
INCOV289151
INCOV289152
INCOV289153
INCOV289154
INCOV289155
INCOV289156
INCOV289157
INCOV289158
INCOV289159
INCOV289160
INCOV289161
INCOV289162
INCOV289163
INCOV289164
INCOV289165
INCOV289166
INCOV289167
INCOV289168
INCOV289169
INCOV289170
INCOV289171
INCOV289172
INCOV289173
INCOV289174
INCOV289175
INCOV289176
INCOV289177
INCOV289178
INCOV289179
INCOV289180
INCOV289181
INCOV289182
INCOV289183
INCOV289184
INCOV289185
INCOV289186
INCOV289187
INCOV289188
INCOV289189
INCOV289190
INCOV289191
INCOV289192
INCOV289193
INCOV289194
INCOV289195
INCOV289196
INCOV289197
INCOV289198
INCOV289199
INCOV289200
INCOV289201
INCOV289202
INCOV289203
INCOV289204
INCOV289205
INCOV289206
INCOV289207
INCOV289208
INCOV289209
INCOV289210
INCOV289211
INCOV289212
INCOV289213
INCOV289214
INCOV289215
INCOV289216
INCOV289217
INCOV289218
INCOV289219
INCOV289220
INCOV289221
INCOV289222
INCOV289223
INCOV289224
INCOV289225
INCOV289226
INCOV289227
INCOV289228
INCOV289229
INCOV289230
INCOV289231
INCOV289232
INCOV289233
INCOV289234
INCOV289235
INCOV289236
INCOV289237
INCOV289238
INCOV289239
INCOV289240
INCOV289241
INCOV289242
INCOV289243
INCOV289244
INCOV289245
INCOV289246
INCOV289247
INCOV289248
INCOV289249
INCOV289250
INCOV289251
INCOV289252
INCOV289253
INCOV289254
INCOV289255
INCOV289256
INCOV289257
INCOV289258
INCOV289259
INCOV289263
INCOV289264
INCOV289265
INCOV289266
INCOV289286
INCOV289287
INCOV289288
INCOV289289
INCOV289291
INCOV289292
INCOV289293
INCOV289294
INCOV289295
INCOV289296
INCOV289297
INCOV289298
INCOV289299
INCOV289300
INCOV289301
INCOV289302
INCOV289303
INCOV289304
INCOV289305
INCOV289306
INCOV289307
INCOV289308
INCOV289309
INCOV289310
INCOV289394
INCOV289395
INCOV289396
INCOV289397
INCOV289398
INCOV289399
INCOV289400
INCOV289401
INCOV289402
INCOV289403
INCOV289404
INCOV289405
INCOV289406
INCOV289407
INCOV289408
INCOV289409
INCOV289410
INCOV289411
INCOV289412
INCOV289413
INCOV289414
INCOV289415
INCOV289416
INCOV289417
INCOV289418
INCOV289419
INCOV289420
INCOV289421
INCOV289422
INCOV289423
INCOV289424
INCOV289425
INCOV289426
INCOV289427
INCOV289428
INCOV289429
INCOV289430
INCOV289431
INCOV289432
INCOV289433
INCOV289434
INCOV289435
INCOV289436
INCOV289437
INCOV289438
INCOV289439
INCOV289440
INCOV289441
INCOV289442
INCOV289443
INCOV289444
INCOV289445
INCOV289446
INCOV289447
INCOV289448
INCOV289449
INCOV289450
INCOV289451
INCOV289452
INCOV289453
INCOV289454
INCOV289455
INCOV289456
INCOV289457
INCOV289458
INCOV289459
INCOV289460
INCOV289461
INCOV289462
INCOV289463
INCOV289464
INCOV289465
INCOV289466
INCOV289467
INCOV289468
INCOV289469
INCOV289470
INCOV289471
INCOV289472
INCOV289473
INCOV289474
INCOV289475
INCOV289476
INCOV289499
INCOV289500
INCOV289501
INCOV289502
INCOV289503
INCOV289504
INCOV289505
INCOV289506
INCOV289507
INCOV289508
INCOV289509
INCOV289510
INCOV289511
INCOV289512
INCOV289513
INCOV289514
INCOV289515
INCOV289516
INCOV289517
INCOV289518
INCOV289519
INCOV289520
INCOV289542
INCOV289543
INCOV289554
INCOV289555
INCOV289556
INCOV289558
INCOV289564
INCOV289565
INCOV289566
INCOV289567
INCOV289568
INCOV289569
INCOV289570
INCOV289571
INCOV289572
INCOV289573
INCOV289574
INCOV289575
INCOV289576
INCOV289577
INCOV289578
INCOV289579
INCOV289580
INCOV289581
INCOV289582
INCOV289583
INCOV289584
INCOV289585
INCOV289586
INCOV289587
INCOV289588
INCOV289589
INCOV289590
INCOV289591
INCOV289592
INCOV289593
INCOV289594
INCOV289595
INCOV289596
INCOV289597
INCOV289598
INCOV289599
INCOV289600
INCOV289601
INCOV289602
INCOV289603
INCOV289604
INCOV289605
INCOV289606
INCOV289607
INCOV289608
INCOV289609
INCOV289610
INCOV289611
INCOV289612
INCOV289613
INCOV289614
INCOV289615
INCOV289616
INCOV289617
INCOV289618
INCOV289619
INCOV289620
INCOV289621
INCOV289622
INCOV289623
INCOV289624
INCOV289625
INCOV289626
INCOV289627
INCOV289628
INCOV289629
INCOV289630
INCOV289631
INCOV289632
INCOV289633
INCOV289634
INCOV289635
INCOV289636
INCOV289637
INCOV289638
INCOV289639
INCOV289640
INCOV289641
INCOV289642
INCOV289643
INCOV289644
INCOV289645
INCOV289646
INCOV289647
INCOV289648
INCOV289649
INCOV289650
INCOV289651
INCOV289652
INCOV289653
INCOV289654
INCOV289655
INCOV289656
INCOV289657
INCOV289658
INCOV289659
INCOV289660
INCOV289661
INCOV289662
INCOV289663
INCOV289664
INCOV289665
INCOV289666
INCOV289667
INCOV289668
INCOV289669
INCOV289670
INCOV289671
INCOV289672
INCOV289673
INCOV289674
INCOV289675
INCOV289676
INCOV289677
INCOV289678
INCOV289679
INCOV289680
INCOV289681
INCOV289683
INCOV289684
INCOV289685
INCOV289686
INCOV289687
INCOV289688
INCOV289689
INCOV289690
INCOV289691
INCOV289692
INCOV289693
INCOV289694
INCOV289695
INCOV289696
INCOV289697
INCOV289698
INCOV289699
INCOV289700
INCOV289701
INCOV289702
INCOV289703
INCOV289704
INCOV289705
INCOV289706
INCOV289707
INCOV289708
INCOV289709
INCOV289710
INCOV289711
INCOV289712
INCOV289713
INCOV289714
INCOV289715
INCOV289716
INCOV289717
INCOV289718
INCOV289719
INCOV289720
INCOV289721
INCOV289722
INCOV289723
INCOV289724
INCOV289725
INCOV289726
INCOV289727
INCOV289728
INCOV289729
INCOV289730
INCOV289731
INCOV289732
INCOV289733
INCOV289734
INCOV289735
INCOV289736
INCOV289737
INCOV289738
INCOV289739
INCOV289740
INCOV289741
INCOV289742
INCOV289743
INCOV289744
INCOV289745
INCOV289746
INCOV289747
INCOV289748
INCOV289749
INCOV289750
INCOV289751
INCOV289752
INCOV289753
INCOV289754
INCOV289755
INCOV289756
INCOV289757
INCOV289758
INCOV289759
INCOV289760
INCOV289761
INCOV289762
INCOV289763
INCOV289764
INCOV289765
INCOV289766
INCOV289767
INCOV289768
INCOV289769
INCOV289770
INCOV289771
INCOV289772
INCOV289773
INCOV289774
INCOV289775
INCOV289776
INCOV289777
INCOV289778
INCOV289779
INCOV289780
INCOV289781
INCOV289782
INCOV289783
INCOV289784
INCOV289785
INCOV289788
INCOV289791
INCOV289793
INCOV289794
INCOV289795
INCOV289796
INCOV289797
INCOV289798
INCOV289799
INCOV289800
INCOV289801
INCOV289802
INCOV289803
INCOV289804
INCOV289805
INCOV289806
INCOV289807
INCOV289808
INCOV289809
INCOV289810
INCOV289811
INCOV289822
INCOV289823
INCOV289824
INCOV289825
INCOV289826
INCOV289827
INCOV289828
INCOV289829
INCOV289830
INCOV289846
INCOV289847
INCOV289848
INCOV289849
INCOV289850
INCOV289851
INCOV289852
INCOV289853
INCOV289897
INCOV289898
INCOV289899
INCOV289900
INCOV289901
INCOV289902
INCOV289904
INCOV289905
INCOV289978
INCOV289983
INCOV289984
INCOV289985
INCOV289986
INCOV289987
INCOV289988
INCOV289989
INCOV289990
INCOV289991
INCOV289993
INCOV289994
INCOV289995
INCOV289996
INCOV289997
INCOV289998
INCOV289999
INCOV290000
INCOV290002
INCOV290004
INCOV290005
INCOV290006
INCOV290007
INCOV290008
INCOV290014
INCOV290015
INCOV290016
INCOV290017
INCOV290018
INCOV290019
INCOV290020
INCOV290021
INCOV290022
INCOV290023
INCOV290024
INCOV290025
INCOV290026
INCOV290027
INCOV290028
INCOV290029
INCOV290030
INCOV290031
INCOV290032
INCOV290033
INCOV290034
INCOV290035
INCOV290036
INCOV290037
INCOV290038
INCOV290039
INCOV290040
INCOV290041
INCOV290042
INCOV290043
INCOV290044
INCOV290045
INCOV290046
INCOV290047
INCOV290048
INCOV290049
INCOV290050
INCOV290051
INCOV290052
INCOV290053
INCOV290054
INCOV290055
INCOV290056
INCOV290057
INCOV290058
INCOV290059
INCOV290060
INCOV290061
INCOV290062
INCOV290063
INCOV290064
INCOV290065
INCOV290066
INCOV290067
INCOV290068
INCOV290069
INCOV290070
INCOV290071
INCOV290072
INCOV290073
INCOV290074
INCOV290075
INCOV290076
INCOV290077
INCOV290078
INCOV290079
INCOV290080
INCOV290081
INCOV290082
INCOV290084
INCOV290086
INCOV290087
INCOV290088
INCOV290089
INCOV290090
INCOV290091
INCOV290092
INCOV290093
INCOV290094
INCOV290095
INCOV290096
INCOV290097
INCOV290098
INCOV290099
INCOV290100
INCOV290101
INCOV290102
INCOV290103
INCOV290104
INCOV290105
INCOV290106
INCOV290107
INCOV290108
INCOV290109
INCOV290110
INCOV290111
INCOV290113
INCOV290114
INCOV290115
INCOV290116
INCOV290117
INCOV290118
INCOV290119
INCOV290120
INCOV290121
INCOV290122
INCOV290123
INCOV290124
INCOV290125
INCOV290126
INCOV290127
INCOV290128
INCOV290129
INCOV290130
INCOV290131
INCOV290132
INCOV290133
INCOV290134
INCOV290135
INCOV290136
INCOV290137
INCOV290138
INCOV290139
INCOV290140
INCOV290141
INCOV290142
INCOV290143
INCOV290144
INCOV290146
INCOV290147
INCOV290148
INCOV290149
INCOV290150
INCOV290151
INCOV290153
INCOV290154
INCOV290155
INCOV290156
INCOV290158
INCOV290159
INCOV290160
INCOV290161
INCOV290162
INCOV290163
INCOV290164
INCOV290165
INCOV290166
INCOV290167
INCOV290168
INCOV290169
INCOV290170
INCOV290171
INCOV290172
INCOV290173
INCOV290174
INCOV290175
INCOV290176
INCOV290177
INCOV290178
INCOV290179
INCOV290180
INCOV290181
INCOV290182
INCOV290183
INCOV290184
INCOV290185
INCOV290186
INCOV290187
INCOV290188
INCOV290189
INCOV290190
INCOV290194
INCOV290195
INCOV290196
INCOV290197
INCOV290198
INCOV290199
INCOV290200
INCOV290201
INCOV290203
INCOV290204
INCOV290206
INCOV290207
INCOV290208
INCOV290209
INCOV290210
INCOV290211
INCOV290213
INCOV290214
INCOV290215
INCOV290216
INCOV290217
INCOV290218
INCOV290219
INCOV290220
INCOV290221
INCOV290222
INCOV290223
INCOV290224
INCOV290225
INCOV290226
INCOV290227
INCOV290228
INCOV290229
INCOV290230
INCOV290231
INCOV290232
INCOV290234
INCOV290235
INCOV290236
INCOV290237
INCOV290238
INCOV290939
INCOV290940
INCOV290941
INCOV290942
INCOV290943
INCOV290944
INCOV290945
INCOV290946
INCOV290947
INCOV290948
INCOV290949
INCOV290950
INCOV290951
INCOV290952
INCOV290953
INCOV290954
INCOV290955
INCOV290956
INCOV290957
INCOV290958
INCOV290959
INCOV290960
INCOV290961
INCOV290962
INCOV290963
INCOV290964
INCOV290965
INCOV290966
INCOV290967
INCOV290968
INCOV290969
INCOV290970
INCOV290971
INCOV290974
INCOV290975
INCOV290976
INCOV290977
INCOV290978
INCOV290979
INCOV290981
INCOV290982
INCOV290983
INCOV290984
INCOV290985
INCOV290986
INCOV290987
INCOV290988
INCOV290989
INCOV290990
INCOV290991
INCOV290992
INCOV290993
INCOV290995
INCOV290996
INCOV290997
INCOV290998
INCOV290999
INCOV291000
INCOV291001
INCOV291002
INCOV291003
INCOV291004
INCOV291005
INCOV291006
INCOV291007
INCOV291008
INCOV291009
INCOV291010
INCOV291011
INCOV291012
INCOV291013
INCOV291014
INCOV291015
INCOV291016
INCOV291017
INCOV291025
INCOV291026
INCOV291032
INCOV291033
INCOV291034
INCOV291035
INCOV291036
INCOV291037
INCOV291038
INCOV291039
INCOV291040
INCOV291041
INCOV291042
INCOV291043
INCOV291044
INCOV291045
INCOV291046
INCOV291047
INCOV291048
INCOV291049
INCOV291050
INCOV291051
INCOV291060
INCOV291061
INCOV291062
INCOV291063
INCOV291064
INCOV291065
INCOV291066
INCOV291067
INCOV291068
INCOV291069
INCOV291070
INCOV291071
INCOV291072
INCOV291073
INCOV291074
INCOV291075
INCOV291076
INCOV291077
INCOV291078
INCOV291079
INCOV291080
INCOV291081
INCOV291082
INCOV291083
INCOV291084
INCOV291085
INCOV291086
INCOV291087
INCOV291088
INCOV291089
INCOV291090
INCOV291091
INCOV291092
INCOV291093
INCOV291094
INCOV291095
INCOV291096
INCOV291097
INCOV291098
INCOV291099
INCOV291100
INCOV291101
INCOV291102
INCOV291103
INCOV291104
INCOV291105
INCOV291106
INCOV291107
INCOV291108
INCOV291109
INCOV291110
INCOV291111
INCOV291112
INCOV291113
INCOV291114
INCOV291115
INCOV291116
INCOV291117
INCOV291118
INCOV291119
INCOV291120
INCOV291121
INCOV291122
INCOV291123
INCOV291124
INCOV291125
INCOV291126
INCOV291127
INCOV291128
INCOV291129
INCOV291130
INCOV291131
INCOV291132
INCOV291133
INCOV291134
INCOV291135
INCOV291136
INCOV291137
INCOV291138
INCOV291139
INCOV291140
INCOV291141
INCOV291142
INCOV291143
INCOV291144
INCOV291145
INCOV291146
INCOV291147
INCOV291148
INCOV291149
INCOV291150
INCOV291151
INCOV291152
INCOV291153
INCOV291154
INCOV291155
INCOV291156
INCOV291157
INCOV291158
INCOV291159
INCOV291160
INCOV291161
INCOV291162
INCOV291163
INCOV291164
INCOV291165
INCOV291166
INCOV291167
INCOV291168
INCOV291169
INCOV291170
INCOV291171
INCOV291172
INCOV291173
INCOV291174
INCOV291175
INCOV291176
INCOV291177
INCOV291178
INCOV291179
INCOV291180
INCOV291181
INCOV291182
INCOV291183
INCOV291184
INCOV291185
INCOV291186
INCOV291187
INCOV291188
INCOV291189
INCOV291190
INCOV291191
INCOV291192
INCOV291193
INCOV291194
INCOV291195
INCOV291196
INCOV291197
INCOV291202
INCOV291203
INCOV291204
INCOV291205
INCOV291206
INCOV291216
INCOV291217
INCOV291218
INCOV291220
INCOV291221
INCOV291222
INCOV291224
INCOV291225
INCOV291314
INCOV291315
INCOV291316
INCOV291317
INCOV291318
INCOV291319
INCOV291320
INCOV291321
INCOV291322
INCOV291323
INCOV291324
INCOV291325
INCOV291326
INCOV291327
INCOV291328
INCOV291329
INCOV291330
INCOV291331
INCOV291332
INCOV291333
INCOV291336
INCOV291341
INCOV291342
INCOV291345
INCOV291346
INCOV291347
INCOV291348
INCOV291349
INCOV291350
INCOV291351
INCOV291352
INCOV291353
INCOV291354
INCOV291355
INCOV291356
INCOV291357
INCOV291358
INCOV291359
INCOV291360
INCOV291361
INCOV291362
INCOV291363
INCOV291364
INCOV291365
INCOV291366
INCOV291367
INCOV291368
INCOV291369
INCOV291370
INCOV291371
INCOV291372
INCOV291373
INCOV291374
INCOV291375
INCOV291376
INCOV291377
INCOV291378
INCOV291379
INCOV291380
INCOV291381
INCOV291382
INCOV291383
INCOV291384
INCOV291385
INCOV291386
INCOV291387
INCOV291388
INCOV291389
INCOV291390
INCOV291391
INCOV291392
INCOV291393
INCOV291394
INCOV291395
INCOV291396
INCOV291397
INCOV291398
INCOV291399
INCOV291405
INCOV291411
INCOV291420
INCOV291422
INCOV291427
INCOV291605
INCOV291606
INCOV291607
INCOV291630
INCOV291631
INCOV291632
INCOV291633
INCOV291634
INCOV291635
INCOV291636
INCOV291639
INCOV291648
INCOV291649
INCOV291650
INCOV291651
INCOV291652
INCOV291653
INCOV291654
INCOV291655
INCOV291656
INCOV291657
INCOV291658
INCOV291659
INCOV291660
INCOV291661
INCOV291662
INCOV291663
INCOV291664
INCOV291665
INCOV291666
INCOV291667
INCOV291668
INCOV291754
INCOV291755
INCOV291756
INCOV291757
INCOV291758
INCOV291759
INCOV291761
INCOV291762
INCOV291781
INCOV291782
INCOV291783
INCOV291784
INCOV291785
INCOV291786
INCOV291787
INCOV291788
INCOV291789
INCOV291794
INCOV291795
INCOV291807
INCOV291808
INCOV291809
INCOV291810
INCOV291811
INCOV291812
INCOV291817
INCOV291823
INCOV291824
INCOV291825
INCOV291826
INCOV291827
INCOV291829
INCOV291831
INCOV291832
INCOV291833
INCOV291837
INCOV291838
INCOV291839
INCOV291855
INCOV291856
INCOV291880
INCOV291889
INCOV291890
INCOV291974
INCOV292069
INCOV292070
INCOV292071
INCOV292078
INCOV292079
INCOV292150
INCOV292157
INCOV292158
INCOV292159
INCOV289887
INCOV289260
INCOV289261
INCOV289262
INCOV289267
INCOV289268
INCOV289269
INCOV289270
INCOV289271
INCOV289272
INCOV289273
INCOV289274
INCOV289275
INCOV289276
INCOV289277
INCOV289278
INCOV289521
INCOV289522
INCOV289540
INCOV289541
INCOV289557
INCOV289682
INCOV289789
INCOV289812
INCOV289813
INCOV289814
INCOV290003
INCOV290145
INCOV290152
INCOV290157
INCOV290191
INCOV290192
INCOV290193
INCOV290212
INCOV291018
INCOV291019
INCOV291020
INCOV291021
INCOV291022
INCOV291023
INCOV291024
INCOV291052
INCOV291053
INCOV291054
INCOV291055
INCOV291198
INCOV291223
INCOV291226
INCOV291313
INCOV291343
INCOV291401
INCOV291402
INCOV291403
INCOV291406
INCOV291642
INCOV291677
INCOV291678
INCOV291679
INCOV291680
INCOV291681
INCOV291682
INCOV291708
INCOV291710
INCOV291711
INCOV291712
INCOV291713
INCOV291714
INCOV291715
INCOV291716
INCOV291760
INCOV291763
INCOV291764
INCOV291765
INCOV291770
INCOV291796
INCOV291797
INCOV291798
INCOV291840
INCOV291857
INCOV291868
INCOV291891
INCOV291920
INCOV291952
INCOV292008
INCOV292009
INCOV292010
INCOV292117
INCOV289280
INCOV289290
INCOV289523
INCOV289786
INCOV289787
INCOV289790
INCOV289815
INCOV289816
INCOV289817
INCOV289818
INCOV289819
INCOV289820
INCOV289821
INCOV289831
INCOV289832
INCOV289833
INCOV289834
INCOV289835
INCOV289836
INCOV289837
INCOV290001
INCOV290205
INCOV290233
INCOV290239
INCOV290240
INCOV290972
INCOV290980
INCOV291056
INCOV291057
INCOV291058
INCOV291059
INCOV291199
INCOV291200
INCOV291207
INCOV291344
INCOV291400
INCOV291404
INCOV291407
INCOV291408
INCOV291410
INCOV291428
INCOV291438
INCOV291637
INCOV291641
INCOV291717
INCOV291718
INCOV291719
INCOV291766
INCOV291767
INCOV291768
INCOV291769
INCOV291776
INCOV291778
INCOV291779
INCOV291790
INCOV291791
INCOV291792
INCOV291820
INCOV291821
INCOV291843
INCOV291858
INCOV291869
INCOV291870
INCOV291882
INCOV291942
INCOV291943
INCOV291956
INCOV292118
INCOV292154
INCOV292155
INCOV289279
INCOV289281
INCOV289282
INCOV289283
INCOV289284
INCOV289285
INCOV289838
INCOV289839
INCOV289840
INCOV289841
INCOV289854
INCOV289855
INCOV289856
INCOV289857
INCOV289858
INCOV289859
INCOV289860
INCOV289893
INCOV289894
INCOV289903
INCOV290009
INCOV290010
INCOV290011
INCOV290973
INCOV291027
INCOV291028
INCOV291029
INCOV291201
INCOV291212
INCOV291213
INCOV291214
INCOV291215
INCOV291219
INCOV291334
INCOV291409
INCOV291413
INCOV291424
INCOV291439
INCOV291638
INCOV291669
INCOV291670
INCOV291720
INCOV291721
INCOV291722
INCOV291723
INCOV291724
INCOV291740
INCOV291771
INCOV291772
INCOV291773
INCOV291774
INCOV291775
INCOV291777
INCOV291780
INCOV291816
INCOV291867
INCOV291871
INCOV291892
INCOV291893
INCOV291921
INCOV291944
INCOV291945
INCOV291979
INCOV291980
INCOV291981
INCOV291982
INCOV291983
INCOV292067
INCOV292072
INCOV292080
INCOV292119
INCOV289524
INCOV289525
INCOV289526
INCOV289527
INCOV289528
INCOV289529
INCOV289530
INCOV289531
INCOV289532
INCOV289533
INCOV289536
INCOV289538
INCOV289539
INCOV289842
INCOV289843
INCOV289844
INCOV289882
INCOV289883
INCOV289884
INCOV289885
INCOV289886
INCOV289888
INCOV289889
INCOV289890
INCOV289891
INCOV290012
INCOV290013
INCOV291030
INCOV291031
INCOV291208
INCOV291209
INCOV291210
INCOV291211
INCOV291412
INCOV291414
INCOV291415
INCOV291416
INCOV291417
INCOV291430
INCOV291538
INCOV291539
INCOV291540
INCOV291541
INCOV291542
INCOV291543
INCOV291544
INCOV291545
INCOV291546
INCOV291547
INCOV291556
INCOV291557
INCOV291558
INCOV291559
INCOV291593
INCOV291594
INCOV291595
INCOV291596
INCOV291643
INCOV291644
INCOV291645
INCOV291646
INCOV291671
INCOV291672
INCOV291673
INCOV291725
INCOV291726
INCOV291727
INCOV291793
INCOV291813
INCOV291828
INCOV291841
INCOV291844
INCOV291864
INCOV291873
INCOV291876
INCOV291883
INCOV291894
INCOV291922
INCOV291935
INCOV291946
INCOV291953
INCOV291957
INCOV291958
INCOV291984
INCOV291985
INCOV292011
INCOV292012
INCOV292036
INCOV292056
INCOV292057
INCOV289845
INCOV289992
INCOV291335
INCOV291418
INCOV291419
INCOV291421
INCOV291423
INCOV291535
INCOV291536
INCOV291537
INCOV291548
INCOV291549
INCOV291561
INCOV291562
INCOV291563
INCOV291564
INCOV291565
INCOV291566
INCOV291567
INCOV291568
INCOV291569
INCOV291577
INCOV291578
INCOV291579
INCOV291580
INCOV291581
INCOV291597
INCOV291598
INCOV291647
INCOV291674
INCOV291675
INCOV291676
INCOV291701
INCOV291702
INCOV291703
INCOV291704
INCOV291705
INCOV291706
INCOV291728
INCOV291729
INCOV291730
INCOV291731
INCOV291732
INCOV291733
INCOV291734
INCOV291735
INCOV291736
INCOV291737
INCOV291738
INCOV291739
INCOV291741
INCOV291742
INCOV291743
INCOV291818
INCOV291830
INCOV291834
INCOV291842
INCOV291851
INCOV291854
INCOV291859
INCOV291860
INCOV291865
INCOV291874
INCOV291878
INCOV291879
INCOV291884
INCOV291923
INCOV291924
INCOV291936
INCOV291937
INCOV291947
INCOV291959
INCOV291960
INCOV291986
INCOV291987
INCOV291988
INCOV292013
INCOV292014
INCOV292015
INCOV292016
INCOV292017
INCOV292018
INCOV292019
INCOV292020
INCOV292065
INCOV292073
INCOV292075
INCOV292081
INCOV292082
INCOV289861
INCOV289862
INCOV289863
INCOV289864
INCOV289865
INCOV289866
INCOV289867
INCOV289868
INCOV289869
INCOV289870
INCOV289871
INCOV289872
INCOV289873
INCOV289874
INCOV289875
INCOV289876
INCOV289877
INCOV289878
INCOV289879
INCOV289880
INCOV289881
INCOV289892
INCOV290994
INCOV291425
INCOV291426
INCOV291433
INCOV291434
INCOV291550
INCOV291551
INCOV291553
INCOV291554
INCOV291555
INCOV291560
INCOV291570
INCOV291571
INCOV291582
INCOV291583
INCOV291584
INCOV291585
INCOV291586
INCOV291587
INCOV291588
INCOV291608
INCOV291640
INCOV291695
INCOV291696
INCOV291697
INCOV291698
INCOV291819
INCOV291835
INCOV291845
INCOV291861
INCOV291881
INCOV291885
INCOV291925
INCOV291926
INCOV291948
INCOV291961
INCOV291989
INCOV291990
INCOV291991
INCOV291992
INCOV292021
INCOV292022
INCOV292023
INCOV292024
INCOV292066
INCOV292074
INCOV292083
INCOV292099
INCOV292100
INCOV292120
INCOV292122
INCOV292123
INCOV292124
INCOV292148
INCOV291436
INCOV291552
INCOV291599
INCOV291836
INCOV291852
INCOV291872
INCOV291875
INCOV291877
INCOV291886
INCOV291899
INCOV291917
INCOV291927
INCOV291928
INCOV291949
INCOV291962
INCOV291993
INCOV292149
INCOV292151
INCOV289544
INCOV289545
INCOV291429
INCOV291431
INCOV291432
INCOV291435
INCOV291437
INCOV291572
INCOV291573
INCOV291574
INCOV291575
INCOV291576
INCOV291589
INCOV291590
INCOV291591
INCOV291592
INCOV291600
INCOV291601
INCOV291602
INCOV291603
INCOV291604
INCOV291610
INCOV291622
INCOV291801
INCOV291814
INCOV291846
INCOV291847
INCOV291848
INCOV291862
INCOV291863
INCOV291866
INCOV291888
INCOV291895
INCOV291896
INCOV291900
INCOV291901
INCOV291902
INCOV291903
INCOV291904
INCOV291908
INCOV291938
INCOV291963
INCOV291975
INCOV291994
INCOV291995
INCOV291996
INCOV292037
INCOV292038
INCOV292039
INCOV292058
INCOV292059
INCOV292060
INCOV292061
INCOV292062
INCOV292063
INCOV292064
INCOV292076
INCOV292216
INCOV292217
INCOV292218
INCOV292219
INCOV292220
INCOV291609
INCOV291611
INCOV291612
INCOV291614
INCOV291623
INCOV291624
INCOV291628
INCOV291629
INCOV291683
INCOV291684
INCOV291685
INCOV291686
INCOV291687
INCOV291688
INCOV291689
INCOV291691
INCOV291692
INCOV291694
INCOV291699
INCOV291707
INCOV291709
INCOV291744
INCOV291752
INCOV291753
INCOV291802
INCOV291849
INCOV291850
INCOV291853
INCOV291887
INCOV291897
INCOV291905
INCOV291906
INCOV291907
INCOV291910
INCOV291929
INCOV291930
INCOV291931
INCOV291939
INCOV291964
INCOV291976
INCOV292025
INCOV292026
INCOV292034
INCOV292040
INCOV292068
INCOV292077
INCOV292098
INCOV292125
INCOV292126
INCOV292135
INCOV292136
INCOV292221
INCOV292222
INCOV292223
INCOV292228
INCOV291613
INCOV291615
INCOV291616
INCOV291617
INCOV291618
INCOV291619
INCOV291620
INCOV291625
INCOV291803
INCOV291898
INCOV291911
INCOV291912
INCOV291918
INCOV291932
INCOV291933
INCOV291940
INCOV291965
INCOV291966
INCOV291997
INCOV291998
INCOV292027
INCOV292028
INCOV292029
INCOV292041
INCOV292042
INCOV292043
INCOV292121
INCOV292127
INCOV292143
INCOV292144
INCOV292145
INCOV292146
INCOV292147
INCOV292152
INCOV292153
INCOV292156
INCOV291626
INCOV291690
INCOV291693
INCOV291700
INCOV291745
INCOV291746
INCOV291804
INCOV291815
INCOV291913
INCOV291914
INCOV291915
INCOV291934
INCOV291950
INCOV291954
INCOV291955
INCOV291967
INCOV291968
INCOV291972
INCOV291973
INCOV291977
INCOV291978
INCOV291999
INCOV292000
INCOV292001
INCOV292002
INCOV292030
INCOV292031
INCOV292044
INCOV292045
INCOV292046
INCOV292047
INCOV292048
INCOV292049
INCOV292101
INCOV292102
INCOV292103
INCOV292104
INCOV292105
INCOV292106
INCOV292107
INCOV291747
INCOV291748
INCOV291805
INCOV291909
INCOV291916
INCOV291941
INCOV291951
INCOV291969
INCOV291970
INCOV291971
INCOV292003
INCOV292004
INCOV292005
INCOV292032
INCOV292033
INCOV292050
INCOV292051
INCOV292108
INCOV292109
INCOV292110
INCOV292111
INCOV292112
INCOV292113
INCOV291621
INCOV291749
INCOV291750
INCOV291751
INCOV291806
INCOV292006
INCOV292007
INCOV292052
INCOV292053
INCOV292054
INCOV291627
INCOV291919
INCOV292114
INCOV292115
INCOV292116
INCOV292128
INCOV292129
INCOV292130
INCOV292131
INCOV292132
INCOV292133
INCOV292134
INCOV292137
INCOV292138
INCOV292139
INCOV292140
INCOV292141
INCOV292035
INCOV292055
INCOV292226
INCOV292227
INCOV292231
INCOV291822
INCOV292142
INCOV292232
INCOV292224
INCOV292229
INCOV292230
INCOV292233
INCOV292225
